# Reconstitution of oral antibiotic suspensions for paediatric use in households: a cross-sectional study among caregivers of 3–5-year-old children from a selected district, Sri Lanka

**DOI:** 10.1186/s12887-024-04725-y

**Published:** 2024-04-04

**Authors:** Malith Kumarasinghe, Manuj C. Weerasinghe

**Affiliations:** Epidemiology Unit, Ministry of Health, No 54/A, New Jayaweera Road, Ethul-Kotte, Sri Jayawardhanapura Kotte, Colombo 10100 Sri Lanka

**Keywords:** Antibiotic, Pediatric, Reconstitutions, Oral suspension, Primary caregiver

## Abstract

**Introduction:**

Reconstitution of oral pediatric antibiotic suspension by primary caregivers plays an essential role in determining the overall health outcome of the child. Incorrect reconstitution techniques could lead to underdosing, overdosing, or introduction of infection. Underdosing could lead to non-resolving infection and antimicrobial resistance.

**Objectives:**

To assess the practice and associated factors on reconstitution of oral pediatric antibiotic suspensions (OPAS) among primary caregivers of 3–5-year-old children in a selected district in Sri Lanka.

**Methods:**

A cross-sectional study was carried out among 835 primary caregivers selected using two-stage cluster sampling at field clinics to assess practices for the reconstitution of OPAS. A live demonstration of the reconstitution of the OPAS was assessed by a checklist. Associated factors with caregiver practices on reconstitution were assessed using Chi-square with the statistical significance level set at 0.05.

**Results:**

A total of 820 respondents were recruited and completed the study (response rate = 98.2%). Overall, 56.0% displayed good performance in the demonstration of reconstitution of oral pediatric antibiotic suspension. Poorest performances were observed in shaking the bottle to loosen the powder (Correct: 53.7%), topping up the bottle with water up to the marked line (Correct: 58.0%), and filling the water below the marked line in the bottle (Correct: 59.0%). Caregivers in urban areas compared to rural and estate regions (45.6% vs. 22.7% and 26.5% respectively) and caregivers aged 35 years or above compared to less than 35 years age group (31.5% vs. 22.5%) performed the reconstitution of OPAS poorly. Parental factors, namely age, gender, level of education, and geographical region (urban/rural/estate) were significantly associated with the performance in reconstituting the oral paediatric antibiotic suspension (*p* = 0.002, *p* < 0.001, *p* < 0.001, and *p* < 0.001 respectively). Factors related to the child, specifically whether they attend preschool and whether they have an older sibling, were found to have a significant association with the correct execution of the reconstitution of OPAS (*p* = 0.017, and *p* = 0.030 respectively).

**Conclusions and recommendations:**

A significant number of primary caregivers displayed poor practice in key steps during the reconstitution of OPAS, which could have a negative impact on the health of the child. Targeted place-based behavioural change health programs with the use of infographic leaflets/ posters may correct the practices of caregivers.

**Supplementary Information:**

The online version contains supplementary material available at 10.1186/s12887-024-04725-y.

## Introduction

Globally, anti-infective medicines are among the most commonly used medicines for young children [1–3]. Therefore, any deficiency in the practice of the reconstitution of these medicines among primary caregivers could have a major negative impact on children [4, 5]. Instructions of the correct reconstitution of antibiotic medicines is usually provided by either the prescriber or the dispenser [6, 7]. Therefore, it is important that the medications for children are prescribed by a qualified medical practitioner and that caregivers buy medications from a qualified pharmacist. Not adhering to the above guidelines in obtaining medicines for children is a major issue worldwide [8].

Correct practice of the reconstitution of antibiotics is critical because, unlike adult medicines, pediatric medicines have many oral dosage forms. Some of them need to be reconstituted or measured with an appropriate device before being administered to a child. Worldwide, it is reported that the most common errors involving pediatric medicines were errors in dosing, misunderstanding of measurement units, mistakes in reconstitution, and storage [9–12]. It is important to understand that prescribing medication for children carries distinctive inaccuracies [13]. For every child, the calculated dosage is different in most circumstances. It depends on children’s weight, age, or body surface area, as well as their preferences [14, 15]. This is a challenging situation for medical prescribers, pharmacists, and other health staff, which needs further exploration [13]. The American Association of Poison Control Centers (AAPCC) has stated that more than half of inquiries to poison centers were regarding children under 5 years old [12]. Childhood medicinal drug overdose is one of the major public health issues that has caused unwanted and unacceptable pediatric injuries worldwide [16]. Therefore, health workers including prescribers and dispensers must provide necessary individualized instructions to raise knowledge about antibiotic suspension usage and to implement appropriate practices.

Overprescription or the use of incorrect doses that are reported at higher prevalence in developing countries nurtures antibiotic resistance [17]. Despite the availability of limited literature in Sri Lanka on antibiotic reconstitution at the household level, the incidence of medicinal errors due to poor practices could be worse. This situation could be exacerbated by the common practice of overprescribing antibiotics [18].

As per the Demographic and Health Survey or DHS (2016), Ratnapura District reported the highest under-5 child mortality (outside Northern and Eastern Provinces) in Sri Lanka [19]. Further, Ratnapura recorded an above-average percentage (2.9%) of acute respiratory infections among under-5 children in the two weeks preceding the 2016/17 DHS [19]. Over 90% of childhood immunization is conducted in government child welfare clinic centers which is the place of recruitment and data collection for this study [20]. Therefore, Ratnapura was selected as the study district.

In summary, studying the reconstitution of oral antibiotic suspensions for pediatric use in households is crucial for ensuring accurate dosing, patient safety, treatment efficacy, and overall success in combating infections while minimizing the risk of antibiotic resistance [17]. It also plays a vital role in empowering caregivers with the knowledge and skills needed for responsible medication administration in pediatric populations [21].

Therefore, this study aims to assess practices, and associated factors on household reconstitution of pediatric oral antibiotic medicines among primary caregivers of 3–5-year-old children in Ratnapura District, Sri Lanka.

## Methods

A descriptive cross-sectional study was conducted among primary caregivers of 3- to 5-year-old children in Ratnapura District of Sri Lanka. Child welfare clinics were selected as the study setting because it was a feasible location for us to approach the study participants in the COVID-19 pandemic era where household surveys were difficult to conduct. Further, more than 90% of the children are immunized in government child welfare clinics thus it provides a representative study sample [20]. Primary caregivers of 3–5-year-old children residing in Ratnapura District for at least 6 months up to the point of data collection were included following informed written consent.

The primary caregiver was defined as the mother/father/relation or the legal guardian who had “the greatest responsibility for the daily care and rearing of a child”. The study recognized both parents as primary caregivers if the child resided with both parents. However, if the child did not reside with either parent, relation, legal guardian, or any other person who primarily provided care and upbringing of the child was considered the primary caregiver, provided he or she was 18 years or over [22]. A two-stage cluster sampling method was used with the selection of Medical Officer of Health (MOH) areas (public health administrative areas in Sri Lanka) as stage one and the selection of child welfare clinic clusters as stage two. Ten MOH areas were randomly selected from the 19 MOH areas in Ratnapura district. A cluster was defined as a single clinic session of a Child Welfare Clinic geographically located within the boundaries of Ratnapura district. The expected proportion of caregivers of 3–5-year-old children with correct practice on reconstitution of oral antibiotic suspension was taken as 0.5 to generate the most conservative sample size with 0.05 taken as the margin of error (Supplementary File 1). Design effect was calculated as 1.9 with cluster size of 10 and rho as 0.1 [23]. Our study population was primary caregivers of 3-5-year-old children from different socio-economic and cultural backgrounds. Hence, there was adequate heterogeneity within clusters that justifies the use of 0.1 as the rho (Supplementary File 1). The calculated sample size was 835, with a 10% nonresponsive rate. The Number of clusters per each selected MOH area was determined using probability proportionate sampling, based on the proportion of 3-to-5-year-old children estimated to be in each selected MOH area (out of the total in the district) for the year 2020. The clusters were selected randomly from a list of all child welfare clinic sessions in each randomly selected MOH area (Detailed sample size calculation-Supplementary File 1).

The study tool consisted of a pretested and validated concise questionnaire and live demonstration by the participant on the reconstitution of oral pediatric antibiotic medicine. Judgmental validity was assessed. The content validity ratio (CVR) and content validity index (CVI) were calculated [24]. All statements reported a CVR of 0.83 or more (one-tailed test *p* = 0.05). Therefore, all statements were retained and taken as CVRt for the calculation of CVI. The CVI for the tool was 0.966, which was acceptable [24].

The questionnaire translated to both Sinhala and Tamil languages, consisted of sociodemographic characteristics, including socioeconomic status, using the Wealth Index (a comprehensive tool and a key indicator used to assess socioeconomic status in the latest Demographic and Health Survey, Sri Lanka) [19].

The interviewer observed the procedure and assessed the participant on predetermined guidelines on correct reconstitution techniques (Supplementary File 2). The principal investigator (PI) and two trained undergraduates of health sciences carried out data collection. Data collectors were given a 2-day training program on the data collection tool, and to reduce interviewer bias and improve the quality of data collection, two interviewer guides were developed by the PI for the “Wealth Index” and the live demonstration of reconstitution of pediatric oral antibiotic suspension. The instructions to the participants were provided in both Sinhala and Tamil languages. The EPICOLLECT5 online platform was used for data collection [25].

### Patient and public involvement

The development of the questionnaire was conducted through consultative meetings with all stakeholders, including the public, and selected mother support group representatives. Mother support groups are an initiative of the Ministry of Health Sri Lanka, to foster the community empowerment of women, and engage women to actively contribute to the positive behavioural changes that may lead to improvements in health and nutrition within communities [26].

### Analysis

Correct practice on reconstitution was given a score with the scoring system, and the decision on the cutoff marks for correct practice was determined by an expert committee (A single expert in each of the areas- pharmacology, sociology, and pediatrics, a practicing medical practitioner, a practicing pharmacist, and a parent of a 3–5-year-old child), taking into consideration literature. Five critical steps were identified from the 10 steps, namely reconstituting an antibiotic suspension, shaking the bottle to loosen the antibiotic powder, filling water to below the line in the bottle, shaking it well, topping up to the line, and selecting the appropriate measuring device. These steps were allocated 2 points for a correct step, zero points for an incomplete step, and (-2) points for an incorrect or not performed step (Criteria for categorization of ‘correct’, ‘incomplete’, and ‘incorrect/not performed’ are detailed in supplementary file 3). For the remaining five steps, 1 point for a correct step, zero points for an incomplete step, and (-1) point for an incorrect or not performed step were allocated. The respondents who scored less than 5 points were assigned to ‘poor’, between 5 and 10 to ‘average’, and above 10 to ‘good’ practice of reconstitution of oral pediatric antibiotic suspension.

Data were initially transferred to MS Excel software from EPICOLLECT5. They were coded and analyzed using Statistical Package for Social Sciences (SPSS) version 22. Rates are presented as proportions. Unadjusted comparisons were carried out using the chi-square test. Statistical significance was calculated based on a p-value of less than 0.05.

### Results

A total of 820 respondents were approached, recruited, and completed the study (response rate = 98.2%). Failure to recruit the total targeted number from the child welfare clinics (*N* = 835) was due to low clinic attendance resulting from the COVID-19 pandemic. Many basic sociodemographic characteristics of our sample of primary caregivers were similar to the parameters of the population in the study location, except having a lower proportion of individuals belonging to Tamil ethnicity and residency in the estate sector (Table 1) (Additional characteristics of the study sample are detailed in Supplementary File 4).

**Table 1:** Comparison of basic characteristics of study respondents in ratnapura district

Basic Characteristics		Study Sample -%	District -%^a^
Ethnicity	Sinhala	90.6	87.1
	Tamil	7.4	10.7
	Muslim	2.0	2.1
Sector/ Geographical region	Rural	84.9	81.7
	Urban	10.9	9.1
	Estate	4.2	9.2
Education	No schooling/Grades 1–5	3.7	5.7
	Grades 6 to 10	22.1	26
	Grade 11 or above	74.3	68.2
Economically active or not	Economically Active	27.9	34.7*
	Economically Inactive	72.1	65.3*

^a^ Out of total population in the district

*As 96.8% of the respondents were females, “economically active or not proportion of females” in the National Census on Population and Housing, 2012 was used instead of proportion out of the total population [27]

The majority of the respondents reported that the mother reconstitutes and administers the medicinal drugs for the child (97.2% and 97%, respectively). Poorly performed steps in the reconstitution of oral pediatric antibiotic suspensions by primary caregivers were shaking the bottle to loosen the antibiotic powder (53.7%), topping up the antibiotic suspension bottle up to the marked line following initial mixing (58%) and initially filling up the antibiotic suspension bottle below the marked line (59%). The satisfactorily performed steps were general steps, such as washing hands (98.3%) and tightly closing the lid of the suspension bottle (97.3%) (Table 2).

**Table 2:** Demonstrated practice of reconstitution of pediatric oral antibiotic suspension by primary caregivers (*N* = 820)

Demonstrated Practice	Correct^a^		Incomplete^a^		Incorrect/ Not performed^a^		Total	
	No	%	No	%	No	%	No	%
Wash hands	806	98.3	7	0.9	7	0.9	820	100.0
Wipe hands on cloth/tissue	772	94.1	39	4.8	9	1.1	820	100.0
Shake the bottle to loosen the powder	440	53.7	61	7.4	319	38.9	820	100.0
Take boiled cooled water	779	95.0	10	1.2	31	3.8	820	100.0
Fill below the line in the bottle	484	59.0	105	12.8	231	28.2	820	100.0
Close the bottle, shake it well	605	73.8	146	17.8	69	8.4	820	100.0
Top up to the line	476	58.0	109	13.3	235	28.7	820	100.0
Select the appropriate measuring device	790	96.3	10	1.2	20	2.4	820	100.0
Take 5 ml to the measuring device*	671	81.8	71	8.7	78	9.5	820	100.0
Tightly close the lid	798	97.3	17	2.1	5	0.6	820	100.0

^*^ Correct- Measured amount within 10% of 5 ml; Incomplete- Measured amount between 10 and 20% of 5 ml; Incorrect- Measured amount more or less than 20% of 5 ml

^a^ Assessment criteria -Supplementary File 3

Overall, 56.0% performed at a “good” level on demonstration of reconstitution of oral pediatric antibiotic suspension, 18.7% at an “average” level, and 25.4% at a “poor” level (Table not provided). Comparatively, a higher proportion of estate sector respondents demonstrated “good” practices than the urban sector respondents (55.9 vs. 34.4%). Similar results were shown for the younger primary caregivers (less than 35 years) on “good” practice than the older respondents (35 years or more) (60.2 vs. 46.9%) (Table 3).

**Table 3:** Distribution of the primary caregivers’ and children’s characteristics according to the demonstrated practice of reconstitution of oral pediatric antibiotic suspension (*N* = 820)

	Number with Good practice (%)	Number with Average practice (%)	Number with Poor practice (%)	Total Number (%)	χ2 (P)
**Primary Caregiver**					
**Sector/ Geographical region**					
Urban	31 (34.4)	18 (20.0)	41 (45.6)	90 (100)	
Rural	409 (58.8)	129 (18.5)	158 (22.7)	696 (100)	26.96 (< 0.001)
Estate	19 (55.9)	6 (17.6)	9 (26.5)	34 (100)	
**Age**					
Below 35 years	337 (60.2)	97 (17.3)	126 (22.5)	560 (100)	12.99 (0.002)
35 & above years	122 (46.9)	56 (21.5)	82 (31.5)	260 (100)	
**Sex**					
Male	5 (19.2)	5 (19.2)	16 (61.5)	26 (100)	20.33 (< 0.001)
Female	454 (57.2)	148 (18.6)	192 (24.2)	794 (100)	
**Ethnic group**					
Sinhala	425 (57.2)	136 (18.3)	182 (24.5)	743 (100)	
Tamil	29 (47.5)	11 (18.0)	21 (34,0.4)	61 (100)	8.24 (0.08)
Sri Lankan Muslim	5 (31.3)	6 (37.5)	5 (31.3)	16 (100)	
Level of education*					
No schooling/grade 1–5	10 (33.3)	7 (23.3)	13 (43.3)	30 (100)	
Grade 6–10	129 (71.3)	21 (11.6)	31 (17.1)	181 (100)	27.16 (< 0.001)
Grade 11 & above	320 (52.5)	125 (20.5)	164 (26.9)	609 (100)	
**Employment status**					
Employed	88 (55.3)	27 (17.0)	44 (27.7)	159 (100)	
Housewife/housework	338 (57.7)	108 (18.4)	140 (23.9)	586 (100)	5.78 (0.216)
Unemployed/retired/student	33 (44.0)	18 (24.0)	24 (32.0)	75 (100)	
**Wealth Index**					
Low	107 (52.2)	41 (20.0)	57 (27.8)	205 (100)	
Middle	227 (55.4)	80 (19.5)	103 (25.1)	410 (100)	3.64 (0.456)
High	125 (61.0)	32 (15.6)	48 (23.4)	205 (100)	
**Child**					
**Age of the child**					
3 years	212(54.5)	79(20.3)	98(25.2)	389(100)	1.377(0.502)
4–5 years	247(57.3)	74(17.2)	110(25.5)	431(100)	
**Child attending preschool**					
No	209(51.4)	89(21.9)	109(26.8)	407(100)	8.185(0.017)
Yes	250(60.5)	64(15.4)	99(24.0)	413(100)	
**Child having an elder sibling**					
No	275(57.1)	76(15.8)	131(27.2)	482(100)	6.995(0.030)
Yes	184(54.4)	77(22.8)	77(22.8)	338(100)	

*Categorization of education- **No schooling/grade 1–5**: No schooling or only attended up to primary school; **Grade 6–10**: Attended up to high school only; **Grade 11 & above**: Attended to College and above [19]

## Discussion

We observed suboptimal performance during critical stages of the reconstitution process, namely the loosening of antibiotic powder before water addition (53.7%), topping up the antibiotic suspension bottle up to the designated line post-initial mixing (58%), and the initial filling of the antibiotic suspension bottle below the marked line (59%). In contrast, participants demonstrated a higher proficiency in general procedures such as handwashing (98.3%), securely sealing the bottle lid (97.3%), selecting the appropriate measuring device (96.3%), using boiled and cooled water (95%), and cleansing hands with a clean cloth/tissue (94.1%). We observed one-fourth of the primary caregivers performed poorly in reconstituting the oral paediatric antibiotic suspension. Gender, sector/ geographical region, education, and age of the primary caregivers and whether the child attends a preschool or not and having an elder sibling or not, were significantly associated with the technique of reconstitution of oral pediatric antibiotic suspension by caregivers whereas socioeconomic status and ethnicity was not.

Few studies globally, have examined the reconstitution of oral liquid medicinal drugs, including dosing measurements. Two distinct study methods can be found in the literature based on the data collection tool. A few studies, including ours, have used live demonstrations of the reconstitution and measuring the doses, whereas a majority have used either an interviewer or respondent-administered questionnaires [9, 21, 28].

Similar to our study, poor practices of reconstitution of suspensions were reported among poor socioeconomic groups [9]. The comparable indicator in our study, the Wealth Index, revealed a pattern consistent with the findings of a study conducted in Paris [9] on reconstitution practices. While the differences were not statistically significant in our study, caregivers with a higher wealth index demonstrated the highest percentage of ‘good practice’ and the lowest percentage of ‘poor practice’ among the three socioeconomic groups (refer to Table 3). The Paris study [9] reported that male caregivers demonstrated a higher likelihood of incorrectly reconstituting the medicine, whereas the likelihood of incorrect reconstitution decreased with the caregiver’s age. Similarly, our study reported that gender was a significant factor associated with the reconstitution of the antibiotic, and male caregivers displayed a 38-percentage point reduction in good practice compared to their female counterparts. However, this result should be interpreted with caution due to the small number of male participants in our study sample. In Sri Lankan culture, the gender perception that females should take care of the child could be the reason for the reduction in male participation in the immunization of children [29]. However, in contrast to the Paris study [9], caregivers in advanced age (35 years or more) displayed a comparatively lower percentage of good practice in our study (13.3-percentage point reduction) [9, 28].

A study in Palestine [28] reported lower usage of correct measuring devices (9.3% used the measuring cup supplied with the medicinal drug) to administer the liquid medicine to the child. However, in our study, 96.3% used the correct measuring device to measure the oral pediatric antibiotic suspension. The major difference between the two studies is the data collection tool, with our study being a live demonstration and the Palestine study [28] using a questionnaire. Despite these differences, primary caregivers in our study showed better use of devices to correctly measure the doses for their children [28]. The use of boiled cooled water for reconstitution is one of the highest correctly performed steps in the reconstitution of our cohort. Similarly, many studies reported a higher proportion of correct use of boiled cool water for reconstitution [30, 31].

Participants from a study conducted in the outpatient clinic of a university hospital in Taiwan [21] performed better than Sri Lankan respondents, with the step of shaking the antibiotic bottle after adding water and topping up the water up to the marked line following shaking displaying the widest gap (our study vs. Taiwan study: 73.8 vs. 98% and 58.0% vs. 72.0%, respectively) [21]. The Taiwan study [21] used a questionnaire to assess the practice. However, the study participants were comparatively well-educated compared to our cohort. Therefore, a reduction in the “correct” proportion of the steps could be expected in our study [21].

Methodologically, an Indian study [31] was the most comparable to our study. The Indian study [31] demonstrated similar parameters in most of the sociodemographic characteristics to the present study [31]. However, key differences were observed in the age of the primary caregiver. Our respondents were older than their Indian counterparts, which is consistent with the difference in the average age of marriage in India and Sri Lanka. The second difference could be observed in the level of education where our study respondents are more educated than their Indian counterparts. Primary caregivers of our study demonstrated a comparatively low level of the correct practice of shaking the bottle against the Indian respondents (Overall as well as caregivers of OPD and in-ward children separately). A lower number of caregivers satisfactorily demonstrated the overall reconstitution process of the medicine in our study, with at least a 15-percentage point reduction compared to the Indian study. The only step in which our respondents performed better was the choice of the reconstitution fluid. 95% of primary caregivers in our study correctly selected boiled cooled water. These findings highlight a concerning fact. Despite better education, our respondents performed comparatively poorly [27, 31].

In our study, primary caregivers who were employed demonstrated suboptimal proficiency in reconstituting oral antibiotic suspension, which was not statistically significant (Table 3). The anticipated improvement in health and nutrition literacy associated with employment and its correlated factors, such as increased income [32], raises questions about the observed lack of improvement in reconstitution practices. A potential contributing factor is the predominance of female respondents (Table 1). It is crucial to approach this insight with caution, considering the low female labour participation rate of 32.5% in Sri Lanka as of the first quarter of 2020 [33]. This demographic aspect necessitates further exploration to discern its impact on health-related practices among employed primary caregivers.

We observed a significant association between education and the practice of reconstruction in our study with a higher proportion of caregivers with limited literacy skills, displaying poor practice (Table 3). Several factors may contribute to the association between low literacy levels, and incorrect practice in preparing oral antibiotic solutions for children. Individuals with lower literacy levels may have had limited access to education, affecting their ability to acquire and retain health-related information, including proper medication preparation [34]. Further, low literacy levels are often associated with limited health literacy, making it challenging for individuals to understand and follow complex healthcare instructions, such as those related to medication preparation [34, 35]. In addition, low literacy can contribute to communication barriers between healthcare providers and individuals with low literacy levels. This may result in a lack of clear understanding of medical instructions, including those related to preparing oral antibiotic solutions [36]. Addressing these issues requires a multifaceted approach, including improving access to education, enhancing health literacy initiatives, providing culturally sensitive healthcare information, and implementing strategies to overcome education barriers in healthcare prescription [37].

Several factors may have contributed to the observation that older mothers (35 years or older), compared to younger mothers, may have poorer practice of the reconstitution of oral antibiotic solutions for their children. Older mothers may have less exposure to recent health education and information, especially if they have not been actively seeking out updated resources. Younger mothers, on the other hand, may have more access to current information through the Internet, social media, and other modern communication channels [38]. Further, older mothers may have received their education during a time when health education and health literacy were not as widespread or emphasized. Further, we have observed widespread initiations to include health literacy in educational curricula in past decades that could have improved the correct practice of young caregivers [39].

Contrary to research suggesting that mothers often acquire more child-rearing experience and knowledge with each subsequent child [40], our study did not confirm this relationship. In fact, lower percentages of both good and poor practices were observed in the reconstitution of OPAS among caregivers of 3–5-year-old children who had an older sibling, compared to those caring for a child without an elder sibling (Table 3).

### Strengths and limitations

The number of study participants that we were able to recruit despite the challenges posed by COVID-19 is a strength of this study while using a robust two-stage cluster sampling technique. Our study used the live demonstration to assess the reconstitution of oral pediatric antibiotic suspension. This method was used to recreate the real-life situation to produce results that are similar to the routine practice by the caregivers. The main disadvantage of the technique is deviating from routine practice when knowingly observed by someone. This could result in an overrepresentation of positive behavior among the study participants known as the Hawthorne effect [41–43]. In contrast, some caregivers might follow the instructions in the medicinal leaflet and reconstitute the antibiotics at home. This cohort might have performed poorly during the demonstration because we did not provide a printed instruction sheet similar to medicinal information leaftet to the participants. Our study revealed a comparatively higher number of estate sector respondents having ‘good’ practices than the urban sector. One possible reason could be the characteristics of the study participants in urban sector, namely the over-representation of low socieconomic groups with limited literacy. Though more than 90% of childhood vaccination takes place in government child welfare clinics in the district [20], the few who opt for private sector immunization could be the higher socioeconomic groups residing in the urban sector. Therefore, our sample might over-represent the poor and individuals with limited literacy skills in the urban sector than the population. The small number of male caregivers in the study sample could have masked any gender-specific characteristic in the reconstitution of the antibiotic suspension. Further, we guided the participants at three specific points during the demonstration of the reconstitution, namely “Please wash your hands appropriately”, “Select the most appropriate water jar to select water for reconstitution”, and “Please take 5 ml of the reconstituted pediatric oral anti-infective suspension to an appropriate measuring device”. In particular, the first two instructions could have resulted in exaggerated positive practice among the primary caregivers by unintentionally encouraging the participants to wash their hands and carefully select the correct type of water (boiled cool water) (Supplementary File 2).

### Study implications

Health professionals including the prescribers and dispensers must approach these observations with sensitivity, recognizing that individual knowledge levels vary widely within any age, ethnicity, and social group. Tailoring medicinal information to specific demographics and considering language differences could improve the reconstitution practices of antibiotics at the household level.

Further, studies are required to observe the routine practice of reconstitution of antibiotics by the caregivers at the household level that expands to administration, storage, and disposal practices at households as well.

## Conclusion

Poor practices in the reconstitution of pediatric oral antibiotic medicines were observed in critical steps of reconstitution. Significant associations were found between the ability to correctly reconstitute oral pediatric antibiotic suspension and certain primary caregiver factors, such as age, gender, education level, and geographical location (urban/rural/estate). Similarly, child-related factors, like attendance at preschool and the presence of an older sibling, were significantly linked to the proper reconstitution of OPAS. Therefore, targeted place-based and culturally adapted programs through mass media and distribution of infographic leaflets on reconstitution instructions in the local language to caregivers when dispensing the drug could be considered to improve the practice. To foster a positive behavioural change in reconstitution practices among primary caregivers, using the mother support groups network could prove to be a valuable resource within the Sri Lankan community. Furthermore, formal training of dispensers on how to provide socio-culturally suitable instructions on reconstitution to caregivers to improve the correct practices could be considered.

### Electronic supplementary material

Below is the link to the electronic supplementary material.

Supplementary Material 1

Supplementary Material 2

Supplementary Material 3

Supplementary Material 4

## Data Availability

Data are available on reasonable request. Raw data without personal identifiers are available from the corresponding author upon reasonable request.

## Declarations

### Disclaimer

The views expressed are those of the authors and not necessarily those of the University of Colombo or the Ministry of Health, Sri Lanka.
