# Supplementary material for: Reconstitution of oral antibiotic suspensions for paediatric use in households: a cross-sectional study among caregivers of 3–5-year-old children from a selected district, Sri Lanka
Source: BMC Pediatr. 2024 Apr 4;24:241. doi: 10.1186/s12887-024-04725-y (PMC10996081; doi:10.1186/s12887-024-04725-y)
Supplement: Supplementary file 1 — Supplementary Material 1 [file 12887_2024_4725_MOESM1_ESM.pdf]

**Supplementary File One**

**Sample size calculation**

$$N = \frac{Z^2 \ P\,(1-P)}{d^2} \qquad (1)$$

N = sample size

D = 0.05 (level of accuracy)

Z = 1.96 (critical value of specified confidence interval)

P = Expected proportion of caregivers of 3–5-year-old children with correct practice on reconstitution of oral antibiotic suspension. A study conducted in Palestine revealed that knowledge on preparation and storage of antibiotic suspensions for paediatrics was around 75 per cent (2). A similar study conducted in Taiwan among paediatric caregivers revealed good knowledge ranging from 46 per cent to 98 per cent depending on the specific aspect of preparation (3). Similar findings were exhibited in a few studies conducted in India with knowledge on preparation, storage and disposal ranging from 50 per cent to 90 per cent. However, Indian studies were mainly focused on adult medications with few questions on accessibility by children (4-6). However, no studies were done to specifically assess the knowledge on preparation, storage and disposal of oral paediatric medicinal drugs of caregivers. Thus, it was decided to use 50 per cent as P.

In primary caregivers of 3-5 year old children, correct practice on reconstitution of paediatric oral antibiotic medications were taken as $P_p$= 50 per cent, $P_s$= 50 per cent and $P_d$= 50 per cent, respectively.

$$N = \frac{1.96^2 \times 0.50 \times (1-0.50)}{0.05^2}$$

N= 384.16 ≈ 385 primary caregivers of 3–5-year-old children

**Definition of a cluster**

A cluster was defined as a single clinic session of a Child Welfare Clinic (CWC) geographically located within boundaries of Ratnapura district.

Rationale behind the decision for selecting a clinic session rather than a Child Welfare clinic was that each clinic session would consists of non-repetitive study units. As care givers of 3-5 years old children was attending Child Welfare clinics for the 3 years MMR (Measles Mumps and Rubella) vaccination and 5-year OPV (Oral Polio vaccine) & DT (Diphtheria and Tetanus) vaccination, there would not be a repetition or overlapping of study units. The cluster was based on vaccination session of a given clinic center and not on geographical area which the clinic center was situated.

On rare occasions, children might be referred to CWC sessions to be assessed by the Medical Officer of Health regarding their nutrition status including children aged 3 to 5 years though Family Health Bureau have instructed to conduct a separate Nutrition clinic for such children. In such instances, they too were included in the study provided that they did not attended a CWC for 3 years vaccination during the period of data collection.

As cluster sampling method was used in this study, correction to the homogeneity within the cluster was added (7).

N= Design effect x n

Design effect = 1+ δ (β-1)

β= cluster size; taken as 10 (as expected average number of caregivers of 3–5-year-old children attending a single child welfare session in Ratnapura District was between 10-15, the cluster size was decided as 10)

δ = rho; in the absence of previous studies, as mentioned by the Bennett and others estimated value of rho was taken as 0.1 (8). The study population was caregivers of 3-5-year-old children. In a given cluster, families of different socio-economic and cultural backgrounds reside. Hence, there was adequate heterogeneity within clusters that justifies the use of 0.1 as the rho.

Design effect = 1+ 0.1 (10-1) =1.9

Therefore, the required sample size= 384.16 x 1.9 = 729.9

≈ 730 primary caregivers of 3–5-year-old children

After adding 10% for non-response,

730 /0.9 = 812

**Sample size was also calculated to accommodate potential associated factors.**

**Table S1- Prevalence of Poor Health Literacy According to Identified Associated Factors among School Teachers in 2017, Colombo District**

| Identified condition / associated factor | P1 | P2 | OR | Sample size |
|---|---|---|---|---|
| Age above 45 | 27.3% | 36.7% | 1.6 | 502 |
| Good knowledge on health | 26.4% | 35.7% | 1.5 | |
| Mass media mode as printed media (newspaper, magazines) accessed to obtain health related information | 28.7% | 40.7% | 1.7 | |

[illegible]

Above sample in Table one, consisted of schoolteachers in Colombo District of Sri Lanka. Therefore, limited/ poor health literacy among general population in Ratnapura District most probably should be more than the percentages reported as in Table 3.7. Therefore, the P1, limited or poor health literacy among the population with the condition or positive for the associated factor was taken as 35 per cent. P2, limited or poor health literacy among the population without the condition or negative for the associated factor was taken as 45 per cent (9).

$$N = \frac{[z_\alpha\sqrt{P(1-P)(1/q_1+1/q_2)} + z_\beta\sqrt{P_1(1-P_1)(1/q_1)+P_2(1-P_2)(1/q_2)}]^2}{(P_1-P_2)^2} \quad (10)$$

P1= 35 per cent

P2= 45 per cent

$Z_{\alpha}$ = Confidence level -95% - 1.96

$Z_{B}$ =Power of 0.8- 0.84

q1= proportion of subjects in group 1- 0.5

q2= proportion of subjects in group 2- 0.5

P= q1P1 + q2P2

N=750.283

N=751

The required total sample size =751; 10% non-response rate= 835

Therefore, the final sample size was taken as 835.

Sample was selected from 10 Medical Officer of Health areas (out of 19 MOH areas) in Ratnapura District. As stated above the cluster size was decided as 10.

The data collection was carried out during the period of 4 months. Usually, 3- to 5-year-old children attend the CWCs to receive vaccination at 3 years (MMR) and 5 years (OPV and DT booster) according to the Expanded Programme of Immunization, Sri Lanka. And their growth monitoring programme is conducted in weighting centres and not in CWCs. Therefore, a child aged 3 to 5 years attending CWCs multiple times in a span of 4 months was highly unlikely. Thus, selecting the clinic session instead of clinic centre is justifiable. Usually, clinic sessions in a given clinic centre was held once a fortnight. However, in some clinic centres sessions are held weekly and in others, centres, once a month.

Number of clusters needed, 835/10 – 84

***The approximate number of clusters available during a single selected month (No. of CWC sessions)***

Number of clinic centres which cater 3–5-year-old children in Ratnapura District - 273

Number of clinic centres which conduct clinics for 3–5-year-old children four times a month - 19

Number of clinic centres which conduct clinics for 3-5 year -old children twice a month - 155

Number of clinic centres which conduct clinics for 3–5-year-old children once a month - 89

**Total number of clinic sessions which cater to 3- 5-year-old children in Ratnapura District (per month)- (19×4) + (155 × 2) + 89 = 475 per month**

Number of clinic sessions for 3 -5 year old children during the period of data collection (4 months) in Ratnapura District- (475×4) = 1900

As we selected only 10 Medical Officer of Health (MOH) areas out of a possible 19 MOH areas, on average, the estimated clinic sessions for the selected MOH areas for the period of data collection (273×4) = 1092 sessions

However, there was the risk of clinic attendance of 3–5-year-old children of less than 10 per session. Therefore additional 10 per cent clusters (9 clusters) were randomly selected as back-up if the desired number was not fulfilled by the initial 84 clusters during the planning stage (total of 93 clinic sessions form 950 clinic sessions).

**Sampling frame**

A list of all CWCs in Ratnapura District was obtained from the Regional Director of Health Services Office. Number of total clusters in the study population would depend on number of clinic sessions per each Child Welfare clinic during the period of data collection which was first 4 months of the year 2021 (January to April).

**Sampling technique**

Two stage cluster sampling method was used.

No of clusters required= 84

However, due to low attendance of children due to the COVID 19 situation in Ratnapura District, in some MOH areas up-to 10 additional clusters were selected randomly (Final number of clusters-128) (In some days, Ratnapura reported the highest number of daily cases out of all districts in Sri Lanka). (Tables S2 & S3).

***First stage***

A list of all MOH areas was prepared. Each MOH area in the list was coded. The list was scrambled, and 10 MOH areas were randomly selected from the list with MS Excel.

1. Ratnapura MC
2. Weligepola
3. Udawalawe (Embilipitiya North)
4. Kuruwita
5. Kiriella
6. Pelmadulla
7. Nivithigala
8. Elapatha
9. Godakawela
10. Kahawatta

***Second stage***

Number of clusters per each selected Medical Officer of Health (MOH) area was determined by the proportion of 3-to-5-year-old children estimated to be in each selected MOH area for the year 2020 using probability proportionate sampling. A list containing number of 3-to-5-year-old children under care, per each selected MOH area for the year 2020 was created for this purpose. Above list was prepared by data obtained from Medical Officer of Maternal & Child Heath, Ratnapura. Following the allocation of number of clusters per each MOH area, selection of clusters within each MOH area was carried out by simple random sampling technique. In each cluster, first ten caregivers that were registered by Public Health Midwives in Clinic Register who meet eligibility criteria was selected for the study.

***Table S2- Sample Size Allocated to Each MOH Area in Ratnapura District***

| | **MOH area** | **3-5yr Children Under Care** | **Number Planned to Select from each MOH** | **Number Selected from each MOH** |
|---|---|---|---|---|
| 1 | Elapatha | 1482 | 51 | 56 |
| 2 | Embilipitiya North (Udawalawe) | 2629 | 89 | 81 |
| 3 | Godakawela | 3469 | 118 | 118 |
| 4 | Kahawatta | 2012 | 69 | 85* |
| 5 | Kiriella | 1278 | 43 | 41 |
| 6 | Kuruwita | 4105 | 140 | 159* |
| 7 | Nivithigala | 2313 | 79 | 62* |
| 8 | Pelmadulla | 3551 | 121 | 89* |
| 9 | Ratnapura MC | 2239 | 77 | 90* |
| 10 | Weligepola | 1420 | 48 | 39 |
| | Total | **24498** | **835** | **820** |

*Due to the COVID 19 pandemic and the lack of human resources (no permanent MOH) and logistic issues, the desired number of respondents were not recruited from Pelmadulla and Nivithigala MOH areas. Therefore, to compensate for the reduced number, a slightly higher number of respondents were recruited from three adjacent MOH areas, namely Ratnapura MC, Kuruwita and Kahawatta.

***Table S3- Number of Clusters Selected in Each MOH Area in Ratnapura District***

| | MOH area | Number of Clinic Sessions Clusters Initially Recruited | Number of Clusters Selected from each MOH |
|---|---|---|---|
| 1 | Elapatha | 6 | 12 |
| 2 | Embilipitiya North (Udawalawe) | 9 | 15 |
| 3 | Godakawela | 12 | 18 |
| 4 | Kahawatta | 7 | 9 |
| 5 | Kiriella | 5 | 8 |
| 6 | Kuruwita | 14 | 25 |
| 7 | Nivithigala | 8 | 8 |
| 8 | Pelmadulla | 13 | 16 |
| 9 | Ratnapura MC | 8 | 11 |
| 10 | Weligepola | 5 | 6 |
| | Total | **87** | **128** |

## References


1. Lwanga SK, Lemeshow S. Sample Size Determination in Health Studies; A Practical Manual. Geneva: World Health Organization; 1991.
2. Al-Ramahi RJ, Zaid AAN, Anabousi H. Problems associated with reconstitution, administration, and storage of antibiotic suspensions for pediatrics: a cross-sectional study in Nablus city, Palestine. BMC Research Notes. 2015;8(1).
3. Hu H, Wu F-LL, Hu F-C, Yang H-Y, Lin S-W, Shen L-J. Effectiveness of Education Programs About Oral Antibiotic Suspensions in Pediatric Outpatient Services. Pediatrics & Neonatology. 2013;54(1):34-42.
4. Sivasankaran P, Mohammed EB, Ganesan N, Durai R. Storage and Safe Disposal of Unwanted/Unused and Expired Medicines: A Descriptive Cross-Sectional Survey among Indian Rural Population. Journal of Young Pharmacists. 2018;11(1):97-100.
5. Sil A, Sengupta C, Das AK, Sil PD, Datta S, Hazra A. A study of knowledge, attitude and practice regarding administration of pediatric dosage forms and allied health literacy of caregivers for children. Journal of Family Medicine and Primary Care. 2017;6(3):636-42.
6. Patel DP, Sneha C, Bhatt SP, S. DS. Storage and Disposal of Medicines In Home Among Students. Journal of Pharmacy Research. 2016;10(6):343-50.
7. Abramson J, Abramson ZH. Research methods in community medicine : surveys, epidemiological research, programme evaluation, clinical trials. 6 ed. New York: Wiley; 2008.

8. Bennett S, Woods T, Liyanage WM, Smith DL. A Simplified general method for cluster-sample surveys of health in developing countries. World Health Statistics Quarterly. 1991;44(3):98-106.
9. Denuwara HMBH, Gunawardena NS. Level of health literacy and factors associated with it among school teachers in an education zone in Colombo, Sri Lanka. BMC Public Health. 2017;17(1).
10. Hulley SB, Cummins SR, Browner WS, Grady DG, Newman TB. Designing Clinical Research. 3 ed: Lippincott Williams & Wilkins; 2007.
