# Supplementary material for: Reconstitution of oral antibiotic suspensions for paediatric use in households: a cross-sectional study among caregivers of 3–5-year-old children from a selected district, Sri Lanka
Source: BMC Pediatr. 2024 Apr 4;24:241. doi: 10.1186/s12887-024-04725-y (PMC10996081; doi:10.1186/s12887-024-04725-y)
Supplement: Supplementary file 2 — Supplementary Material 2 [file 12887_2024_4725_MOESM2_ESM.pdf]

Reconstitution of oral antibiotic suspensions for paediatric use in households: A cross-sectional study among caregivers of 3–5-year-old children in a selected district, Sri Lanka

Questionnaire

1.0 IDENTIFICATION

Serial Number			
MOH area:			
PHM area:			
Name of the Clinic:			
Sector (Urban/Rural/Estate)			

Please underline the appropriate answer

1.1 Who is the main carer of the child at home in given four time slots?

Weekdays		Weekends	
Daytime (6am-6pm)	Night (6pm-6am)	Daytime (6am-6pm)	Night (6pm-6am)
Mother<br>Father<br>Grandparent<br>Relative<br>Maid<br>Other (sp.)<br>__________	Mother<br>Father<br>Grandparent<br>Relative<br>Maid<br>Other (sp.)<br>__________	Mother<br>Father<br>Grandparent<br>Relative<br>Maid<br>Other (sp.)<br>___________	Mother<br>Father<br>Grandparent<br>Relative<br>Maid<br>Other (sp.)<br>__________

1.2 Most of the time, who administer medicines to your child?

Mother
Father
Grandparent
Relative
Maid
Other (specify) _____________

1.3 Most of the time, who prepare medicines to your child?

Mother
Father
Grandparent
Relative
Maid
Other (specify) ____________

1.4 Your Relationship to the child

Mother
Father
Grand parents
Brother/ Sister
Relative
Legal guardian
Other (specify) ____________

1.5 Age of the child: Years | | | Months | | | Days | | |

1.6 Does your child attend a pre-school?
Yes
No

1.7 Do your chid attend day-care?
Day-care only
After preschool day care
Creche
No

1.8 Does the child have elder sibling
Yes
No

2.0 DEMOGRAPHIC AND SOCIO-ECONOMIC DETAILS

2.1 How old are you at 1st of May, 2019

Years [ ][ ] Months [ ][ ] Days [ ][ ]

Please tick in the correct box

2.2 What is your sex? Male [ ] Female [ ]

2.3 Which ethnicity do you belong?

Sinhala	
Sri Lanka Tamil	
Indian Tamil	
Sri Lanka Moor	
Other (specify) _____________	

2.4 What is your highest Educational level?

No formal schooling	
Grade1-5; Not completed Grade 5 (Some primary education)	
Completed grade 5 (Completed primary)	
Grade6-10; Not completed Grade 10 (Some secondary education)	
Completed grade 10 (Completed secondary education)	
Grade 11 or higher (Higher than secondary education)	

2.5 What is your current paid employment?

If employed, tick the appropriate employment group and skip to Section 3.0

Legislators, senior officials and managers	
Professionals	
Technicians and associate professionals	
Clerks	
Service workers and shop and market sales workers	
Skilled agricultural and fishery workers	
Craft and related trades workers	
Plant and machine operators and assemblers	
Elementary occupations	
Armed forces and others	
Self-employed	
Not employed	

2.6 If you are not employed, what are you doing at present?

Student	
Home maker	
Retired	
Unemployed (able to work)	

3.0 HOUSEHOLD AND HOUSING CHARACTERISTICS (wealth Index)

3.1 Main source of drinking water and water for cooking for member of your household

Source	Drinking water	Source	Water for cooking
Protected well		Protected well	
Semi-protected well		Semi-protected well	
Unprotected well		Unprotected well	
Tap within unit		Tap within unit	
Tap within premises but outside unit		Tap within premises but outside unit	
Tap outside premises		Tap outside premises	
Rural water supply project		Rural water supply project	
Tube well		Tube well	
Bowser		Bowser	
River/Tank/Streams/Spring		River/Tank/Streams/Spring	
Rain water		Rain water	
Bottle water		Bottle water	
Other (specify):		Other (specify):	

3.2 What kind of toilet facility do member of your household usually use?

Flush to piped sewer system	
Flush to septic tank	
Flush to pit latrine	
Flush to somewhere else	
Flush don’t know where	
Ventilated improved pit latrine	
Pit latrine - with slab	

3.3 Do you use cooker or stove for cooking usually?

Cooker	
Stove	

3.4 What is the main source of fuel used in your household for cooking?

Electricity	
Gas (LP)	
Kerosene	
Saw dust/Rice husk/Charcoal	
Other (Specify):	

3.5 What is the main source of lighting for your household?

National grid electricity	
Rural Hydro power electricity	
Kerosene	
Solar power	
Other (Specify):	

3.6 Principal materials of construction of the housing unit

A. Roof		B. Walls		C. Floor	
Tile		Brick		Cement	
Asbestos		Cement block/stone		Terrazzo/Tile/Granite	
Concrete		Cabook		Mud	
Zink Aluminum sheet		Pressed soil brick		Wood	
Metal sheet		Mud		Sand	
Cadjan/Palmyrah/Straw		Cadjan/Palmyrah		Concrete	
Other (specify)		Plank/Metal sheet		Other (specify)	
		Other (specify)			

3.7 Does your household have following items/sources?

Electricity	
Solar power	
Watch/clock	
Radio	
Television	
Mobile telephone	
Telephone land	
Refrigerator	
Computer	
Washing machine	
Rice cooker	

3.8 Does any member of this household own,

Bicycle	
Motorcycle/ scooter	
Trishaw	
Tractor/ land master	
Car/Van/Jeep	
Bus/Lorry/Truck	

3.9 The house you live in

Owned by a household member	
Government owned	
Private owned (rent/ lease)	
Occupied free of rent	
Encroached	
Other (specify)	
Domestic staff	

Interviewer Guide on Observation of Preparation of paediatric oral anti-infective suspensions

This will be a practical station

There will be 3 substations for the practical

1) Washing hands
2) Preparation of paediatric oral anti-infective suspension
3) Taking 5ml to an of the reconstituted paediatric oral anti-infective suspension appropriate measuring cup

1. Washing hands

Please complete 4.3.1 and 4.3.2 sections of the check list after observation

Command to participants: "Please wash your hands appropriately"

- If a working tap with a sink is available within the premises, station should be located near the sink.
- Soap should be placed near the sink
- If no sink or tap is available, use the empty basin and water bawl provided for you. Please place the soap near the empty basin with the water bawl.
- Please refer visual aid 1 for correct technique of hand washing.
- Time allocated for this activity is 30 seconds
- Please request the participant to move to next station as soon as time expires.

2. **Preparation of paediatric oral anti-infective suspension**

Please complete 4.3.3 to 4.3.7 sections of the check list after observation

Command to participants- "Select the most appropriate water jar to select water for reconstitution"

- There will be 3 water jars.
  - Tap cool water
  - Boiling water
  - Boiled cooled water

- Near these 3 jars there will be paediatric oral anti-infective suspension bottle for the activity
- Time allocated for this activity is 1 minute.
- Please request the participant to move to next station as soon as time expires.

3. Taking 5ml of the reconstituted paediatric oral anti-infective suspension to an appropriate measuring cup

Please complete 4.3.8 to 4.3.10 sections of the check list after observation

Command to participants: "Please take 5ml of the reconstituted paediatric oral anti-infective suspension to an appropriate measuring device"

- There will be following measuring devices.
- Measuring cup
- Table spoon
- Dropper

- Lid of a paediatric oral anti-infective suspension bottle
- Time allocated for this activity is 30 seconds.
- Following 30 seconds please request the participant to stop the activity.

Preparation of paediatric oral anti-infective suspensions and fill the following Checklist

No	Observation	Correct	Incomplete	Incorrect
1	Wash the hands with clean water and soap			
2	Wipe the hands with clean cloth			
3	Shake the bottle to loosen the powder			
4	Take water- boiled cooled water			
5	Fill the bottle with water to just below the line marked in the bottle or label			
6	Close the lid and invert the bottle shake well			
7	Top up with boiled cooled water up-to the line marked in the bottle or label			
8	Selecting the appropriate measuring device			
9	Open the bottle and take 5ml to the selected appropriate measuring device (Measuring cup)			
10	Tightly close the lid			
