# Supplementary material for: Reconstitution of oral antibiotic suspensions for paediatric use in households: a cross-sectional study among caregivers of 3–5-year-old children from a selected district, Sri Lanka
Source: BMC Pediatr. 2024 Apr 4;24:241. doi: 10.1186/s12887-024-04725-y (PMC10996081; doi:10.1186/s12887-024-04725-y)
Supplement: Supplementary file 3 — Supplementary Material 3 [file 12887_2024_4725_MOESM3_ESM.pdf]

Supplementary File Three- Assessment Criteria for the Checklist

Table S4- Assessemnt Criteria for the Checklist

	**Demonstrated Practice**	**Correct**	**Incomplete**	**Incorrect/ Not performed**
1	Wash hands	CDC Five steps in hand washing- First four steps<br>1. Wet your hands with clean, running water (warm or cold), turn off the tap, and apply soap.<br>2. Lather your hands by rubbing them together with the soap. Lather the backs of your hands, between your fingers, and under your nails.<br>3. Scrub your hands for at least 20 seconds. Need a timer? Hum the "Happy Birthday" song from beginning to end twice.<br>4. Rinse your hands well under clean, running water.	Only a single step is missed except the step: The use of soap/ hand wash.	If the order is not followed or if soap/ hand wash is not used.
2	Wipe hands on cloth/tissue	Dry your hands using a clean towel (CDC's fifth step in hand washing)	If water remains in hands after drying	Did not dry or wipe the hands using inappropriate cloth (e.g., own trousers)
3	Shake the bottle to loosen the powder	Shaking the bottle holding the bottle in between the two fingers (from the top and bottle of the bottle)	Only rolling horizontally placing it between the palms of the hands	Did not do
4	Take boiled cooled water	Taking the boiled cooled water after reading the label in the bottle	Taking the boiled cooled water without properly reading the label in the bottle	Did not take the boiled cooled water
5	Fill below the line in the bottle	Fill about two-thirds below the line in the bottle	Filled close to the marked line in the bottle	Filled over the line or filled below half away from the marked line in the bottle

6	Close the bottle, shake it well	Shaking the bottle vigorously holding the bottle in between two fingers (from the top and bottle of the bottle) for at least 5 seconds (count to five)	Either did not shake the bottle vigorously holding the bottle by the two fingers (from the top and bottle of the bottle) or did not shake at least 5 seconds (count to five)	Did not shake at all or only rolled horizontally between palms of the hands
7	Top up to the line	Filled up to the line with 5% or less (or more) of the 100ml (This was performed after the participant completed the demonstration by separately calculating the amount in the measurement device (by a pipette) and the amount in the bottle)	Between 5% to 10%	More than 10%
8	Select the appropriate measuring device	Selection of measuring cup	Selection of a different measure device and then changing over to the correct device at the start of the step 9	Wrong measuring device and attempting to take 5 ml from the selected device
9	Take 5 ml to the measuring device	Measured amount within 10 percent of 5 ml,	Measured amount between 10 and 20 percent of 5 ml	Measured amount more or less than 20 percent of 5 ml.
10	Tightly close the lid	Lid is closed tightly	The lid id not tight after closing	Not closely the lid
