# Supplementary material for: Reconstitution of oral antibiotic suspensions for paediatric use in households: a cross-sectional study among caregivers of 3–5-year-old children from a selected district, Sri Lanka
Source: BMC Pediatr. 2024 Apr 4;24:241. doi: 10.1186/s12887-024-04725-y (PMC10996081; doi:10.1186/s12887-024-04725-y)
Supplement: Supplementary file 4 — Supplementary Material 4 [file 12887_2024_4725_MOESM4_ESM.pdf]

Supplementary File 4- Socioeconomic characteristics of the primary caregivers

Table S5- Distribution of Respondents According to the Sector (N=820)

MOH area	Sector							
	Urban		Rural		Estate		Total	
	No	%	No	%	No	%	No	%
Elapatha	0	0.0	55	98.2	1	1.8	56	100.0
Godakawela	0	0.0	106	89.8	12	10.2	118	100.0
Kahawatta	0	0.0	77	90.6	8	9.4	85	100.0
Kiriella	0	0.0	39	95.1	2	4.9	41	100.0
Kuruwita	0	0.0	157	98.7	2	1.3	159	100.0
Nivithigala	0	0.0	59	95.1	3	4.9	62	100.0
Pelmadulla	0	0.0	84	94.4	5	5.6	89	100.0
Ratnapura MC	90	100.0	0	0.0	0	0.0	90	100.0
Udawalawe	0	0.0	80	98.7	1	1.3	81	100.0
Weligepola	0	0.0	39	100.0	0	0.0	39	100.0
**Total**	**90**	**100.0**	**696**	**100.0**	**34**	**100.0**	**820**	**100.0**

MOH area- Medical Officer of Health area

Table S6- Distribution of Respondents According to the Level of Education (N=820)

MOH area	Level of Education							
	No Schooling/Grade 1-5		Grade 6-10		Grade 11 & Above		Total	
	No	%	No	%	No	%	No	%
Elapatha	1	1.8	5	8.9	50	89.3	56	100.0
Godakawela	4	3.4	39	33.1	75	63.6	118	100.0
Kahawatta	5	5.9	23	27.1	57	67.1	85	100.0
Kiriella	1	2.4	2	4.9	38	92.7	41	100.0
Kuruwita	4	2.5	8	5.0	147	92.5	159	100.0
Nivithigala	6	9.7	6	9.7	50	80.6	62	100.0
Pelmadulla	3	3.4	32	36.0	54	60.7	89	100.0
Ratnapura MC	4	4.4	9	10.0	77	85.6	90	100.0
Udawalawe	2	2.5	42	51.9	37	45.7	81	100.0
Weligepola	0	0.0	15	48.5	24	61.5	39	100.0
**Total**	**30**	**3.7**	**181**	**22.1**	**609**	**74.3**	**820**	**100.0**

MOH area- Medical Officer of Health area

Table S7- Employment Status and Current Employment of the Respondents According to the Sector (N=820)

Variable	Sector							
	Urban		Rural		Estate		Total	
	No	%	No	%	No	%	No	%
**Employment status**								
Employed	15	16.7	124	17.8	20	58.8	159	19.4
Housewife/housework	61	67.8	514	73.9	11	32.4	586	71.5
Retired/student	0	0.0	5	0.7	0	0.0	5	0.6
Unemployed	14	15.6	53	7.6	3	8.8	70	8.5
Total	90	100.0	696	100.0	34	100.0	820	100.0
**Employment***								
Armed forces and others	0	0.0	4	3.2	0	0.0	4	2.5
Clerks	0	0.0	12	9.7	0	0.0	12	7.5
Craft and related trades workers	1	6.7	9	7.3	1	5.0	11	6.9
Elementary occupations	3	20.0	18	14.5	19	95.0	40	25.2
Legislators, senior officials and managers	3	20.0	18	14.5	0	0.0	21	13.2
Plant and machine operators and assemblers	0	0.0	2	1.6	0	0.0	2	1.3
Professionals	5	33.3	43	34.7	0	0.0	48	30.2
Service workers and shop and market sales workers	3	20.0	14	11.3	0	0.0	17	10.7
Skilled agricultural and fishery workers	0	0.0	2	1.6	0	0.0	2	1.3
Technicians and associate professionals	0	0.0	2	1.6	0	0.0	2	1.3
**Total**	**15**	**100.0**	**124**	**100.0**	**20**	**100.0**	**159**	**100.0**

*Classification of the employment was based on the ISCO-08 occupation categorisation
